# Pharmacovigilance assessment of gout: a real-world study using the FAERS database

**DOI:** 10.3389/fphar.2026.1868887

**Published:** 2026-07-08

**Authors:** Honghao Ren, Nannan Yao, Xiaodong Ren, Yani Su, Hongjun Yao, Jing Hu, Pengfei Wen, Ming Zhang, Peng Xu, Weikun Hou, Zhi Yang, Mingyi Yang

**Affiliations:** 1 Department of Joint Surgery, HongHui Hospital, Xi’an Jiaotong University, Xi’an, Shaanxi, China; 2 Xi’an Key Laboratory of Pathogenesis and Precision Treatment of Arthritis, Xi’an, Shaanxi, China; 3 Department of Radiotherapy, Tangdu Hospital, The Fourth Military Medical University, Xi’an, Shaanxi, China; 4 Department of General Practice, Honghui Hospital, Xi’an Jiaotong University, Xi’an, Shaanxi, China

**Keywords:** disproportionality analysis, FAERS, gout, pharmacovigilance, real-world study

## Abstract

**Objective:**

Drug intervention is a key method for preventing gout, various drugs have been implicated as potential risk factors in individual studies. This study aims to comprehensively identify drugs linked to the development of gout.

**Methods:**

Data were obtained from the FDA Adverse Event Reporting System (FAERS), and disproportionality analysis was employed to quantitatively assess the associations between drugs and gout. Four complementary signal detection methods—Reporting Odds Ratio (ROR), Proportional Reporting Ratio (PRR), Bayesian Confidence Propagation Neural Network (BCPNN), and Multi-item Gamma Poisson Shrinker (MGPS)—were utilized. To further delineate exposure–outcome relationships and identify influential predictors, least absolute shrinkage and selection operator (LASSO) logistic regression was implemented. Time-to-onset (TTO) analysis was conducted to examine the temporal dynamics between drug initiation and the occurrence of gout. Finally, a comprehensive assessment of the therapeutic indications of the drugs was performed.

**Results:**

A total of 35 drugs were ultimately identified as potentially associated with the onset and progression of gout. Among these, several agents have been previously reported in the literature as having possible links to gout development. In addition, a number of novel candidates were detected for which evidence of an association with gout remains limited or has not been clearly established. These include Lenalidomide, Sacubitril valsartan, Ruxolitinib, Treprostinil, Octreotide, Selexipag, Rosuvastatin, Sitagliptin, Riociguat, Epoprostenol, Patiromer, Dasabuvir ombitasvir paritaprevir ritonavir, Tafamidis, Sparsentan, and Iloprost. Furthermore, TTO analysis suggested that approximately 75% of gout events occurred within 0.6 years following initiation of therapy. These pharmacotherapeutic agents are employed across diverse clinical settings, encompassing haematological malignancies, cardiovascular diseases, and pulmonary hypertension.

**Conclusion:**

These findings suggest the potential for targeted monitoring of drug-associated gout in clinical practice. When administering these medications, it may be crucial to regularly assess patients’ uric acid levels and maintain heightened awareness for the possible onset of gout.

## 1 Introduction

Gout is a condition resulting from the deposition of monosodium urate crystals in articular and non-articular structures, with elevated serum urate concentration being the most important risk factor for its development (Dalbeth et al., 2021). The typical initial presentation is an intensely painful acute inflammatory arthritis (gout flare) affecting a lower limb joint, characterized by unpredictable pain, joint swelling, redness, and cumulative joint damage from recurrent flares. Beyond these clinical symptoms, gout is often associated with serious complications, including hyperuricemia, chronic kidney disease, premature death, and various metabolic diseases (Shi et al., 2023). As dietary intake of purine-rich foods has increased alongside rising material living standards, the prevalence of gout has risen annually (Singh et al., 2020). The central strategy for effective gout management is long-term urate-lowering therapy to reverse hyperuricaemia, which promotes the dissolution of monosodium urate crystals and prevents long-term gout flares (Dalbeth et al., 2021). Despite advances in understanding the pathophysiology of gout and the availability of effective urate-lowering therapies, management remains suboptimal (Sims, 2019). Currently, no curative treatment exists worldwide; therefore, long-term prevention represents the most important aspect of clinical gout management (Robinson, 2018).

Achieving sustained prevention of gout flares is a critical clinical objective, with long-term urate-lowering therapy serving as the primary strategy for this purpose (Sims, 2019). Equally essential is the mitigation of associated risk factors, which include hyperuricemia, age, sex, body weight, dietary habits, and alcohol intake (Neogi, 2016). Beyond these, various pharmacologic agents may induce hyperuricemia and subsequently elevate gout risk. For instance, diuretics promote hyperuricemia through enhanced renal urate reabsorption, reduced renal urate excretion, and volume contraction. Low-dose aspirin increases risk via urate-retaining effects on the kidneys, whereas high-dose aspirin exhibits uricosuric properties. Additional medications that impair renal urate clearance encompass cyclosporine, pyrazinamide, and ethambutol (Neogi, 2016). Although pharmacotherapy is central to the prevention of gout, and certain drugs have been implicated as potential risk factors in individual studies, a comprehensive characterization of medications associated with the onset of gout remains incomplete.

**FIGURE 1:**
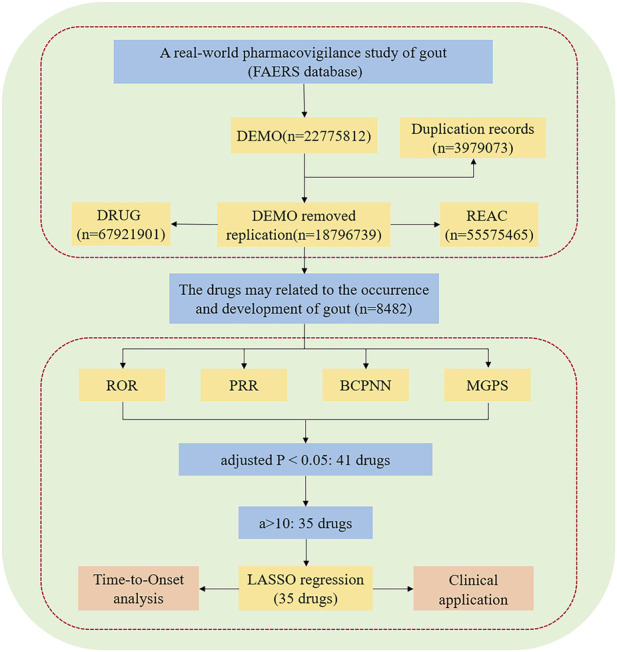
The flowchart of this study.

Within the context of drug safety evaluation, real-world data serve not only as a critical resource but also as a valuable adjunct to post-marketing surveillance. Among existing spontaneous reporting systems, the FDA Adverse Event Reporting System (FAERS) stands as the most extensive and comprehensive database globally. Owing to its large sample sizes and prolonged observation periods, FAERS facilitates the early identification of rare or serious adverse event signals (Wen et al., 2026). Consequently, this system has been widely adopted for signal detection and ongoing pharmacovigilance (Li et al., 2026; Roberto et al., 2026; Yan et al., 2026). In the current investigation, we leveraged reports from the FAERS database to pinpoint medications associated with gout attacks. These findings are expected to support the development of targeted interventions aimed at mitigating gout risk in clinical practice and may potentially reduce the occurrence of drug-induced gout. To enhance methodological transparency and promote clearer comprehension of the study design, a schematic diagram outlining the primary workflow is provided in Figure 1.

## 2 Materials and methods

### 2.1 Data acquisition and deduplication

The data source for this investigation was the FAERS, covering the interval from the first quarter of 2004 through the first quarter of 2025. Each record within FAERS is organized into seven standardized data modules: DEMO (containing demographic and administrative metadata, such as unique case identifiers), REAC (adverse events expressed using MedDRA Preferred Terms), DRUG (medications involved in the reported events), OUTC (patient outcomes), RPSR (source of the report), THER (therapeutic schedules and durations), and INDI (MedDRA-coded indications for drug use). Following FDA-recommended deduplication guidelines, a hierarchical retention approach was applied: for identical CASE IDs, the most current record according to the fda_dt field was kept; for entries sharing both the same CASE ID and fda_dt, the record with the highest PRIMARYID was retained. Any remaining duplicate entries were then subjected to an additional deduplication step. Adverse events were classified using MedDRA Preferred Terms (PTs), with gout designated as the primary PT of interest. With regard to drug–event associations, each listed agent was assigned a suspected role—Primary Suspect, Secondary Suspect, Concomitant, or Interacting. Only those medications categorized as Primary Suspect were included in the subsequent exposure–outcome analyses.

### 2.2 Disproportionality analysis

To quantitatively assess drug–adverse event relationships within the FAERS database, a disproportionality analysis was conducted using four complementary signal detection algorithms: Reporting Odds Ratio (ROR), Proportional Reporting Ratio (PRR), Bayesian Confidence Propagation Neural Network (BCPNN), and Multi-item Gamma Poisson Shrinker (MGPS) (Wang et al., 2025; Qi et al., 2025). For the ROR approach, the criteria were defined as a ≥ 3 and a lower bound of the 95% confidence interval (CI) exceeding 1. The PRR method applied thresholds of PRR ≥ 2 along with a ≥ 3. The BCPNN algorithm employed an IC025 (Information Component 2.5th percentile) greater than 0. For the MGPS analysis, an EB05 (Empirical Bayes 5th percentile) > 2 was adopted (Hong et al., 2025). To improve the specificity and robustness of signal identification, only those drug–event pairs that simultaneously satisfied all four criteria were retained for subsequent evaluation. During the initial screening phase, two-sided P-values were calculated for each candidate pair. Given the multiplicity challenge inherent in high-dimensional pharmacovigilance data, these P-values were then adjusted using a false discovery rate (FDR)-controlling procedure, with statistical significance defined as a two-sided adjusted P-value < 0.05.

### 2.3 LASSO logistic regression

To further elucidate the exposure–outcome relationship and to identify the most parsimonious set of predictive variables, the Least Absolute Shrinkage and Selection Operator (LASSO) logistic regression technique was applied. Initially, drug candidates were prioritized based on the frequency of gout-related adverse events derived from the disproportionality analysis. Only those agents with a reported frequency exceeding 10 for gout events were retained for subsequent LASSO regression modeling. To optimize model performance and prevent overfitting, the optimal penalty parameter (λ) was determined via cross-validation, thereby enabling effective variable selection and regularization.

### 2.4 Time-to-onset analysis

To investigate the temporal relationship between pharmacotherapy initiation and the subsequent clinical presentation of gout, a systematic time-to-onset (TTO) analysis was undertaken. In this study, TTO was operationally defined as the elapsed time from the commencement of treatment (START_DT) to the documented date of adverse event occurrence (EVENT_DT), thereby providing a quantitative basis for assessing the chronological sequence linking drug exposure to the development of gout. This temporal measure enabled the identification of specific time intervals characterized by heightened susceptibility to adverse events following drug administration. Consequently, it supports the delineation of high-risk windows during the post-treatment phase, offering valuable insights for targeted clinical monitoring and risk mitigation strategies.

### 2.5 Pharmacovigilance clinical indications

To clarify the clinical disease profiles associated with medications linked to gout events, a comprehensive evaluation of therapeutic indications was carried out based on data retrieved from the FAERS database. This analysis aims to further specify which underlying medical conditions—when managed with particular pharmacological agents—might facilitate the initiation or exacerbate the progression of gout. The findings obtained from this investigation are anticipated to assist in the optimization of clinical disease management frameworks, encourage appropriate prescribing practices, and strengthen both the prevention and treatment of gout-related comorbidities.

### 2.6 Statistical analysis

All analytical workflows and statistical calculations were executed using the R software environment (version 4.5.0). A significance threshold of adjusted P < 0.05 was predefined as the criterion for statistical significance, and this standard was consistently maintained across all inferential tests performed throughout the study.

## 3 Results

### 3.1 Data acquisition results

**FIGURE 2:**
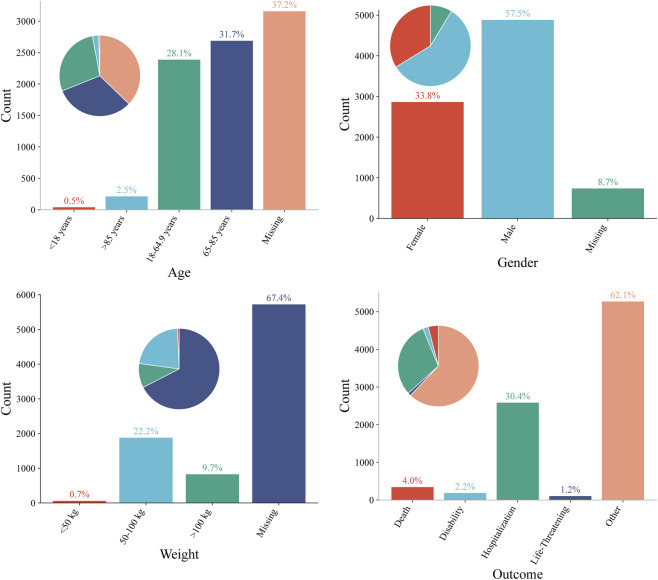
Basic information of gout patients related to drug use, including age, gender, weight and outcomes.

Query of the FAERS database across the period from the first quarter of 2004 through the first quarter of 2025 identified a total of 8,482 individual case safety reports describing gout events potentially related to pharmacotherapeutic exposure. Stratification by age revealed that adults (18–64.9 years) and elderly individuals (65–85 years) accounted for the largest proportions of reports, representing 28.1% and 37.1% of cases, respectively. By contrast, pediatric patients (under 18 years) and the very elderly (over 85 years) comprised only 0.5% and 2.5% of the cohort, respectively. Assessment of demographic characteristics indicated a pronounced male predominance, with male patients representing 57.5% of the cohort compared with 33.8% for female patients. With respect to body weight distribution, the 50–100 kg range constituted the most frequently observed stratum (22.2%), whereas underweight individuals (below 50 kg) and those with obesity (exceeding 100 kg) accounted for smaller proportions (0.7% and 9.7%, respectively). Evaluation of clinical outcomes demonstrated that hospitalization was the most commonly reported serious adverse event (30.4%), followed by death (4.0%), disability (2.2%), and life-threatening conditions (1.2%). The majority of reports (62.1%) fell under the “other' outcome designation (Figure 2).

### 3.2 Disproportionality analysis

**FIGURE 3:**
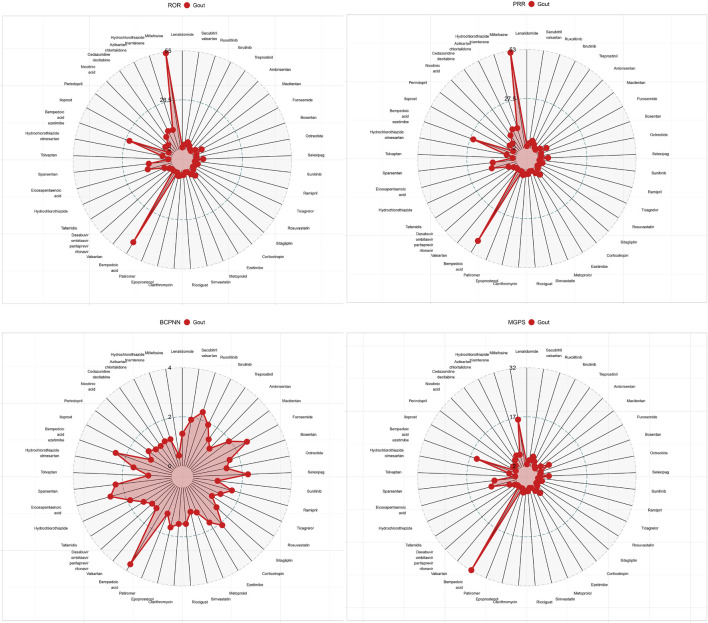
Disproportionality analysis: ROR, PRR, BCPNN and MGPS.

A total of 41 pharmaceutical agents simultaneously satisfied the criteria across all four signal detection algorithms—namely ROR, PRR, BCPNN, and MGPS—while also meeting the adjusted statistical significance threshold of P < 0.05. These drugs are as follows: Lenalidomide, Sacubitril valsartan, Ruxolitinib, Ibrutinib, Treprostinil, Ambrisentan, Macitentan, Furosemide, Bosentan, Octreotide, Selexipag, Sunitinib, Ramipril, Ticagrelor, Rosuvastatin, Sitagliptin, Corticotropin, Ezetimibe, Metoprolol, Simvastatin, Riociguat, Clarithromycin, Epoprostenol, Patiromer, Bempedoic acid, Valsartan, Dasabuvir ombitasvir paritaprevir ritonavir, Tafamidis, Hydrochlorothiazide, Eicosapentaenoic acid, Sparsentan, Tolvaptan, Hydrochlorothiazide olmesartan, Bempedoic acid ezetimibe, Iloprost, Perindopril, Nicotinic acid, Cedazuridine decitabine, Azilsartan chlortalidone, Hydrochlorothiazide triamterene and Miltefosine (Figure 3, Table 1).

**TABLE 1:** The results of disproportionality analysis.

Drugs	a	ROR	PRR	BCPNN	MGPS	P value	adj-P value
		Cl (95%Cl)	PRR (χ2)	IC (IC025)	EBGM (EBGM05)		
Lenalidomide	486	2.85 (2.60–3.12)	2.84 (548.11)	1.45 (1.31)	2.74 (2.50)	7.90E-121	5.60E-118
Sacubitril valsartan	210	4.48 (3.90–5.14)	4.47 (551.19)	2.13 (1.91)	4.38 (3.82)	3.80E-121	2.70E-118
Ruxolitinib	194	6.08 (5.27–7.01)	6.05 (800.23)	2.57 (2.32)	5.94 (5.15)	5.7E-175	4.10E-172
Ibrutinib	144	4.65 (3.94–5.48)	4.63 (403.74)	2.19 (1.92)	4.57 (3.88)	5.07E-89	3.60E-86
Treprostinil	135	3.28 (2.76–3.89)	3.27 (209.61)	1.69 (1.42)	3.23 (2.73)	5.15E-47	3.66E-44
Ambrisentan	114	2.74 (2.27–3.29)	2.73 (123.66)	1.44 (1.15)	2.71 (2.25)	2.37E-28	1.69E-25
Macitentan	101	4.91 (4.03–5.98)	4.89 (309.52)	2.28 (1.94)	4.85 (3.98)	1.91E-68	1.36E-65
Furosemide	86	7.97 (6.44–9.86)	7.92 (515.18)	2.97 (2.55)	7.85 (6.34)	1.50E-112	1.00E-109
Bosentan	57	4.19 (3.22–5.43)	4.17 (136.77)	2.05 (1.60)	4.15 (3.20)	6.59E-31	4.68E-28
Octreotide	44	3.79 (2.81–5.09)	3.78 (89.44)	1.91 (1.39)	3.76 (2.80)	1.27E-20	9.01E-18
Selexipag	41	7.42 (5.46–10.10)	7.38 (225.33)	2.88 (2.23)	7.35 (5.40)	1.48E-49	1.05E-46
Sunitinib	40	2.87 (2.10–3.92)	2.87 (48.43)	1.51 (1.00)	2.86 (2.09)	8.77E-12	6.23E-09
Ramipril	38	4.77 (3.47–6.57)	4.76 (112.36)	2.25 (1.65)	4.74 (3.44)	1.95E-25	1.38E-22
Ticagrelor	38	3.54 (2.57–4.87)	3.53 (68.64)	1.81 (1.26)	3.52 (2.56)	4.21E-16	2.99E-13
Rosuvastatin	38	2.89 (2.10–3.98)	2.89 (46.77)	1.53 (0.99)	2.88 (2.09)	2.07E-11	1.47E-08
Sitagliptin	37	4.02 (2.91–5.56)	4.01 (83.41)	2.00 (1.42)	4.00 (2.89)	3.00E-19	2.13E-16
Corticotropin	35	7.13 (5.11–9.95)	7.10 (182.68)	2.82 (2.11)	7.07 (5.07)	1.43E-18	1.02E-15
Ezetimibe	35	5.16 (3.70–7.19)	5.14 (116.23)	2.36 (1.72)	5.12 (3.67)	3.33E-26	2.36E-23
Metoprolol	35	3.25 (2.33–4.53)	3.24 (54.07)	1.69 (1.12)	3.23 (2.32)	5.98E-13	4.24E-10
Simvastatin	33	3.04 (2.16–4.28)	3.03 (44.84)	1.60 (1.02)	3.03 (2.15)	5.97E-11	4.24E-08
Riociguat	32	4.38 (3.09–6.20)	4.37 (82.77)	2.12 (1.48)	4.35 (3.07)	4.96E-19	3.52E-16
Clarithromycin	31	4.43 (3.11–6.31)	4.42 (81.75)	2.14 (1.48)	4.41 (3.09)	8.53E-19	6.06E-16
Epoprostenol	31	5.15 (3.61–7.33)	5.13 (102.76)	2.35 (1.67)	5.11 (3.59)	2.97E-23	2.11E-20
Patiromer	30	3.49 (2.44–5.00)	3.48 (52.97)	1.80 (1.17)	3.47 (2.43)	1.18E-12	8.39E-10
Bempedoic acid	30	47.85 (33.20–68.97)	46.06 (1,318.74)	5.52 (3.70)	45.90 (31.84)	3.21E-39	2.28E-36
Valsartan	30	3.63 (2.53–5.19)	3.62 (56.70)	1.85 (1.21)	3.61 (2.52)	1.89E-13	1.34E-10
Dasabuvir ombitasvir paritaprevir ritonavir	29	3.61 (2.50–5.20)	3.60 (54.29)	1.84 (1.20)	3.59 (2.49)	6.39E-13	4.54E-10
Tafamidis	25	4.57 (3.09–6.78)	4.56 (69.32)	2.19 (1.43)	4.55 (3.07)	4.96E-16	3.52E-13
Hydrochlorothiazide	24	6.79 (4.54–10.15)	6.76 (117.54)	2.75 (1.88)	6.74 (4.51)	8.50E-13	6.03E-10
Eicosapentaenoic acid	21	15.44 (10.03–23.75)	15.25 (279.22)	3.93 (2.59)	15.22 (9.89)	3.98E-18	2.83E-15
Sparsentan	16	14.32 (8.74–23.45)	14.16 (195.52)	3.82 (2.29)	14.14 (8.63)	1.09E-13	7.77E-11
Tolvaptan	15	3.72 (2.24–6.17)	3.71 (29.63)	1.89 (0.94)	3.70 (2.23)	2.26E-05	0.016054
Hydrochlorothiazide olmesartan	14	7.06 (4.17–11.95)	7.03 (72.31)	2.81 (1.58)	7.02 (4.15)	2.75E-08	1.95E-05
Bempedoic acid ezetimibe	13	26.42 (15.24–45.8)	25.87 (310.64)	4.69 (2.44)	25.84 (14.91)	1.17E-14	8.33E-12
Iloprost	12	4.46 (2.53–7.86)	4.44 (32.01)	2.15 (1.01)	4.44 (2.52)	2.63E-05	0.018680
Perindopril	9	7.64 (3.96–14.71)	7.59 (51.53)	2.92 (1.28)	7.59 (3.94)	4.33E-06	0.003075
Nicotinic acid	8	7.13 (3.56–14.29)	7.10 (41.89)	2.83 (1.11)	7.09 (3.54)	2.36E-05	0.016791
Cedazuridine decitabine	6	11.20 (5.01–25.02)	11.10 (55.16)	3.47 (1.09)	11.09 (4.96)	2.15E-05	0.015237
Azilsartan chlortalidone	6	13.44 (6.01–30.06)	13.3 (68.27)	3.73 (1.17)	13.29 (5.94)	7.79E-06	0.005531
Hydrochlorothiazide triamterene	6	13.05 (5.84–29.18)	12.92 (65.99)	3.69 (1.16)	12.91 (5.77)	9.18E-06	0.006521
Miltefosine	3	54.35 (17.09–172.91)	52.03 (150.23)	5.70 (0.43)	52.02 (16.35)	2.93E-05	0.020838

### 3.3 LASSO logistic regression

**FIGURE 4:**
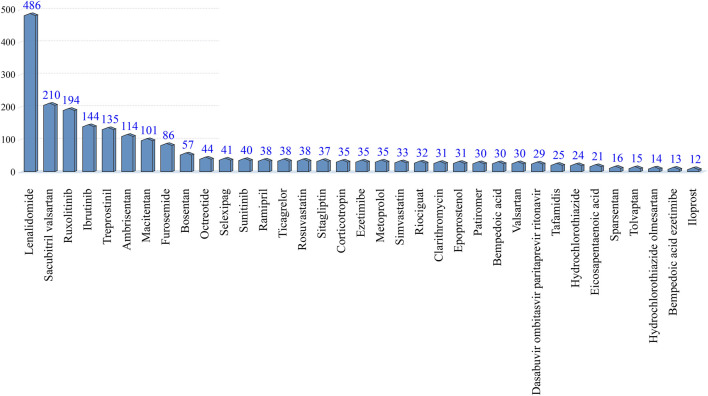
The 35 drugs with a frequency exceeding 10.

**FIGURE 5:**
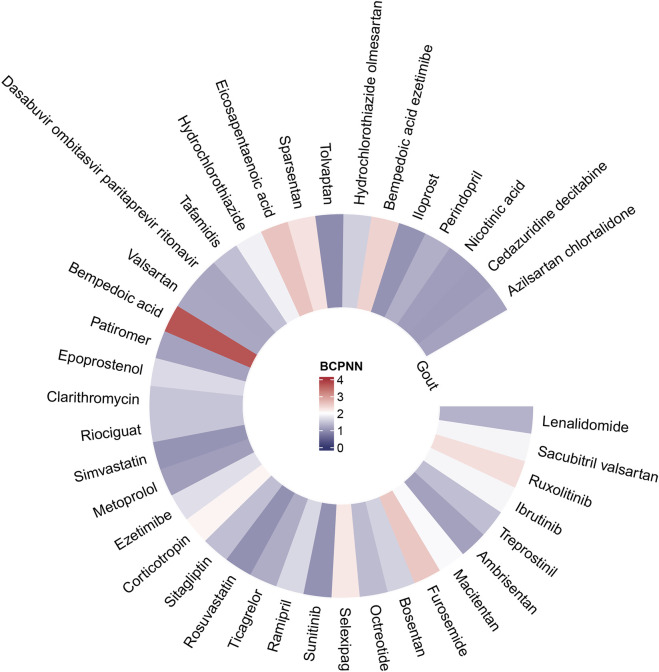
The drugs obtained through LASSO regression analysis.

**FIGURE 6:**
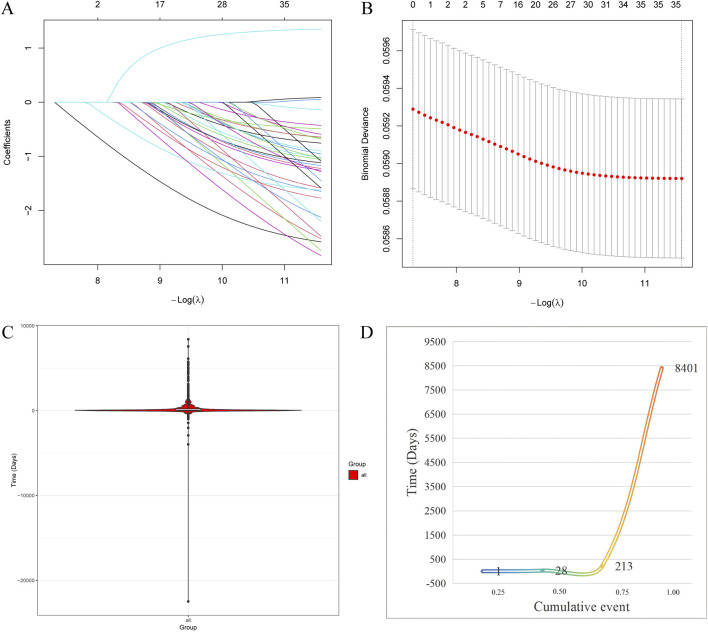
LASSO regression analysis and TTO analysis. **(A,B)** LASSO regression analysis; **(C,D)** TTO analysis.

From the 41 drugs enumerated above, a subset of 35 agents with a frequency exceeding 10 (a > 10) was selected for the construction of a LASSO regression model (Figure 4). All 35 drugs yielded non-zero coefficients in the fitted model. The estimated coefficients are presented as follows: Lenalidomide (−0.0046), Sacubitril valsartan (−0.0045), Ruxolitinib (−0.0046), Ibrutinib (−0.0040), Treprostinil (−0.0040), Ambrisentan (−0.0035), Macitentan (0.0004), Furosemide (−0.0039), Bosentan (−0.0024), Octreotide (−0.0030), Selexipag (−0.0031), Sunitinib (−0.0046), Ramipril (−0.0026), Ticagrelor (−0.0035), Rosuvastatin (−0.0019), Sitagliptin (0.0003), Corticotropin (0.0138), Ezetimibe (−0.0017), Metoprolol (−0.0033), Simvastatin (−0.0041), Riociguat (−0.0032), Clarithromycin (−0.0034), Epoprostenol (−0.0006), Patiromer (0.0022), Bempedoic acid (−0.0039), Valsartan (−0.0023), Dasabuvir ombitasvir paritaprevir ritonavir (−0.0032), Tafamidis (−0.0043), Hydrochlorothiazide (−0.0029), Eicosapentaenoic acid (−0.0036), Sparsentan (−0.0033), Tolvaptan (−0.0045), Hydrochlorothiazide olmesartan (−0.0024), Bempedoic acid ezetimibe (−0.0038), and Iloprost (−0.0043). The model selected on the basis of the minimum cross-validation error (λ.min) retained all 35 drugs without exclusion (Figures 5, 6A,B).

### 3.4 Time-to-onset analysis

An analysis of latency periods was conducted to explore the temporal relationship between the commencement of pharmacological treatment and the emergence of gout. Examination of the TTO data revealed a pronounced right-skewed distribution, characterized by a median latency of 28 days and an interquartile range spanning 213 days. This pattern indicates that approximately 75% of gout episodes were reported within the first 0.6 years following initiation of therapy, while the remaining cases manifested over a considerably more extended period. Such variability underscores notable inter-individual differences in vulnerability to drug-induced gout (Figures 6C,D).

### 3.5 Pharmacovigilance clinical indications

**FIGURE 7:**
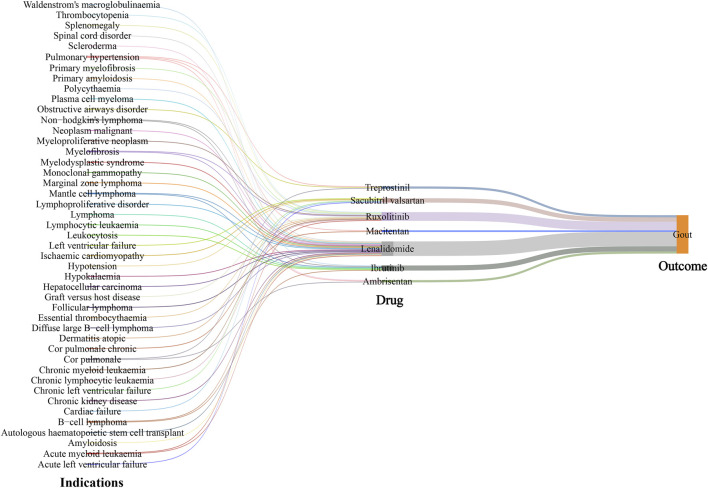
Clinical indications for the 7 drugs with a frequency greater than 100.

Based on the 35 candidate agents identified through the aforementioned LASSO regression analysis, we further conducted a structured review of their approved and commonly reported clinical indications, with particular emphasis on the seven drugs exhibiting a frequency greater than 100. The main clinical indications of Lenalidomide include:acute myeloid leukaemia, amyloidosis, autologous haematopoietic stem cell transplant, B-cell lymphoma, chronic kidney disease, diffuse large B-cell lymphoma, follicular lymphoma, hepatocellular carcinoma, hypokalaemia, lymphoproliferative disorder, mantle cell lymphoma, marginal zone lymphoma, monoclonal gammopathy, myelodysplastic syndrome, myelofibrosis, neoplasm malignant, non-hodgkin’s lymphoma, plasma cell myeloma, primary amyloidosis and spinal cord disorder. The main clinical indications of Sacubitril valsartan include:acute left ventricular failure, cardiac failure, chronic left ventricular failure, hypotension, ischaemic cardiomyopathy and left ventricular failure. The main clinical indications of Ruxolitinib include:acute myeloid leukaemia, chronic lymphocytic leukaemia, chronic myeloid leukaemia, dermatitis atopic, essential thrombocythaemia, graft versus host disease, myelofibrosis, myeloproliferative neoplasm, polycythaemia, primary myelofibrosis, splenomegaly and thrombocytopenia. The main clinical indications of Ibrutinib include:B-cell lymphoma, leukocytosis, lymphocytic leukaemia, lymphoma, mantle cell lymphoma, non-hodgkin’s lymphoma and waldenstrom’s macroglobulinaemia. The main clinical indications of Treprostinil include:cor pulmonale, obstructive airways disorder and pulmonary hypertension. The main clinical indications of Ambrisentan include:cor pulmonale, pulmonary hypertension and scleroderma. The main clinical indications of Macitentan include:cor pulmonale chronic and pulmonary hypertension (Figure 7).

## 4 Discussion

This study utilized data from the FAERS database to identify medications potentially associated with gout. Following a comprehensive series of analyses, we identified 35 distinct drugs that may contribute to the onset and progression of gout. Several of these medications have been previously implicated in gout development in prior research, including Ibrutinib, Ambrisentan, Macitentan, Furosemide, Bosentan, Sunitinib, Ramipril, Ticagrelor, Corticotropin, Ezetimibe, Metoprolol, Simvastatin, Clarithromycin, Bempedoic acid, Valsartan, Hydrochlorothiazide, Eicosapentaenoic acid, Tolvaptan, and Hydrochlorothiazide olmesartan. Additionally, we identified a number of drugs for which the association with gout remains unclear or underexplored. These novel drugs include Lenalidomide, Sacubitril valsartan, Ruxolitinib, Treprostinil, Octreotide, Selexipag, Rosuvastatin, Sitagliptin, Riociguat, Epoprostenol, Patiromer, Dasabuvir ombitasvir paritaprevir ritonavir, Tafamidis, Sparsentan, and Iloprost. The findings from this study suggest that, when these drugs are prescribed in clinical practice, it may be necessary to monitor uric acid levels and remain vigilant for the potential development of gout.

Zheng et al. also utilized the FAERS database to assess drug-induced hyperuricemia and gout, identifying 131 drugs associated with hyperuricemia and 177 with gout. While both studies share a common data source and topic, there are several fundamental differences that distinguish our study and provide unique value (Zheng et al., 2025). First, our study employed four complementary signal detection methods (ROR, PRR, BCPNN, and MGPS), whereas Zheng et al. used three methods (ROR, PRR, BCPNN). The inclusion of MGPS in our analysis offers enhanced stability in sparse data scenarios and reduces false-positive signals through empirical Bayesian shrinkage, thereby increasing the robustness of the identified associations. Second, we focused exclusively on gout as the clinical endpoint, whereas Zheng et al. combined hyperuricemia and gout. Gout represents a symptomatic inflammatory arthritis with direct patient impact, whereas hyperuricemia is often an asymptomatic biochemical abnormality. This focused approach yields more clinically actionable insights. Third, a key innovation of our study is the systematic emphasis on 16 previously unrecognized or poorly characterized drug candidates (e.g., Lenalidomide, Sacubitril valsartan, Ruxolitinib, Selexipag, Rosuvastatin, Sitagliptin, Tafamidis, and Sparsentan) that have limited or no prior evidence linking them to gout. While Zheng et al. listed many drugs, they did not specifically highlight these agents as novel or under-recognized. Fourth, our time-to-onset analysis revealed that approximately 75% of gout events occurred within 0.6 years (approximately 7.2 months) after drug initiation, providing a clinically meaningful surveillance window. This temporal profile differs from the median 31 days reported by Zheng et al., likely due to differences in drug composition and case definition. Finally, we translated our findings into direct, practical recommendations for clinical monitoring—specifically, regular uric acid level assessments and heightened vigilance for gout onset when administering the identified medications. This translational emphasis is less developed in Zheng et al.‘s primarily descriptive and methodological report. In summary, while Zheng et al. provided a broad screening of drug-induced hyperuricemia and gout, our study offers methodological enhancement (incorporation of MGPS), a focused gout-specific endpoint, discovery of 16 novel drug candidates, a distinct time-to-onset profile, and actionable clinical guidance. These unique features collectively justify the novelty and contribution of our work to the existing literature.

Acute gout flares affecting the bilateral first metatarsophalangeal joints have been reported in patients with chronic lymphocytic leukemia receiving Ibrutinib (Kaur et al., 2021). Prior investigations have identified novel adverse drug reaction signals for endothelin receptor antagonists—including Ambrisentan, Macitentan, and Bosentan—through disproportionality analysis, implicating a potential link with gout (Han et al., 2026). Diuretics such as Furosemide contribute to hyperuricemia by increasing renal uric acid reabsorption, decreasing its excretion, and inducing fluid contraction, thereby facilitating the onset and progression of gout (Neogi, 2016). In patients with advanced renal cell carcinoma, gout was observed during the third week of treatment with Temsirolimus and Sunitinib Malate (Patel et al., 2009). A comprehensive analysis integrating FAERS data with Mendelian Randomization (MR) indicates a significant causal association between hypertension and gout risk. While the lower bounds of the 95% confidence intervals for the ROR of candesartan, lisinopril, and ramipril all exceed 1—suggesting a potential relationship with gout—these findings do not establish a direct causal effect of these antihypertensive agents on gout development (He et al., 2025). Ticagrelor has been associated with a higher incidence of in-hospital gout compared with clopidogrel among patients with acute coronary syndrome (Liu et al., 2025). Gout induced by ticagrelor is likely an underrecognized adverse effect during prolonged therapy. As a reversible P2Y12 receptor antagonist, ticagrelor is rapidly absorbed and metabolized, with its primary metabolite AR-C124910XX displaying comparable potency but some pharmacokinetic differences. Unlike drugs primarily metabolized by the liver, ticagrelor is predominantly processed via the kidneys, and elevated concentrations in renal blood may impair renal function, thereby disrupting uric acid filtration, absorption, uptake, and excretion (Zhang et al., 2015).

The relationship between Corticotropin and gout remains somewhat contentious. While the majority of studies suggest that Corticotropin is effective in managing acute gout, some reports indicate that adrenocorticotropic hormone also exerts therapeutic effects. These effects are thought to be mediated through two mechanisms: firstly, by stimulating the adrenal glands to secrete glucocorticoids, and secondly, by rapidly inhibiting the activation of peripheral white blood cells via the melatonin receptor 3 signaling pathway (Cronstein and Terkeltaub, 2006). However, other research points to the potential of ACTH in triggering the onset of acute gouty arthritis (Hellman, 1949). Colchicine, which is widely approved for the treatment of gout and familial Mediterranean fever, may encounter interactions with certain drugs. For instance, Clarithromycin, a potent inhibitor of CYP3A4 and P-glycoprotein, significantly extends the half-life of colchicine, thereby increasing the risk of adverse reactions such as diarrhea, pancytopenia, bone marrow failure, and vomiting when used concurrently (Sundy, 2010). The use of Ezetimibe in patients with hyperlipidemia has been linked to new cases of hyperuricemia, where blood uric acid levels exceed 7.0 mg/dL (Su et al., 2026). In addition, Metoprolol has been shown to elevate uric acid levels and increase the risk of gout in African American patients with chronic kidney disease due to hypertension (Juraschek et al., 2017). Similarly, the combination of Ezetimibe/Simvastatin and sustained-release niacin in individuals with diabetes or metabolic syndrome and hyperlipidemia leads to increased uric acid levels (Fazio et al., 2010).

Bempedoic acid represents an emerging lipid-lowering therapy. Enhanced cholesterol control achieved through Bempedoic acid administration is associated with a decreased risk of major adverse cardiovascular events, including myocardial infarction and coronary revascularization. The overall safety profile of the drug is acceptable, although an elevated risk of gout has been reported (De Filippo et al., 2023). In patients intolerant to statins, Bempedoic acid treatment was linked to a reduced incidence of major adverse cardiovascular events, defined as cardiovascular death, nonfatal myocardial infarction, nonfatal stroke, or coronary revascularization. Nevertheless, compared with placebo, Bempedoic acid use was accompanied by increased occurrences of gout and cholelithiasis, as well as mild elevations in serum creatinine, uric acid, and hepatic enzyme levels (Nissen et al., 2023). Investigations have indicated that Valsartan exerts either a neutral or slightly urate-lowering effect on serum uric acid (Sutton Burke et al., 2019). Thiazide diuretics, commonly employed to prevent recurrent nephrolithiasis, can, when administered as Hydrochlorothiazide, result in elevated uric acid concentrations in patients with kidney stones (Dhayat et al., 2023). Moreover, previous studies have demonstrated that dietary intake of Eicosapentaenoic acid interacts with genetic predisposition to modulate gout risk (Chen et al., 2025). Tolvaptan, a selective vasopressin V2 receptor antagonist, has gained attention as a potential therapy for autosomal dominant polycystic kidney disease; however, its use may be associated with hyperuricemia and gout due to reduced renal urate excretion (Bellos, 2021). Finally, in patients with systolic hypertension treated with a combination of Hydrochlorothiazide and Olmesartan, serum uric acid levels increased in a dose-dependent manner, although no acute gout flares were observed (Joseph et al., 2007).

Published clinical evidence substantiates the association between several drugs identified in our analysis and gout or hyperuricemia. Ibrutinib has been linked to acute gout flares in chronic lymphocytic leukemia patients. Endothelin receptor antagonists (Ambrisentan, Macitentan, Bosentan) have previously shown gout signals via disproportionality analysis. Loop and thiazide diuretics (Furosemide, Hydrochlorothiazide) are well-established causes of hyperuricemia. Ticagrelor is associated with increased in-hospital gout compared with clopidogrel. Bempedoic acid consistently raises gout risk in clinical trials, whereas Valsartan appears urate-neutral or slightly urate-lowering. Corticotropin has dual effects—therapeutic in acute gout but also reported to trigger attacks. Ezetimibe, Metoprolol, and Tolvaptan have been implicated in hyperuricemia or gout. Collectively, these published reports provide external validation for many of the signals identified in our FAERS-based analysis, reinforcing the clinical relevance and credibility of our findings.

It should be acknowledged that the seven most frequently reported drugs in this study (Lenalidomide, Sacubitril valsartan, Ruxolitinib, Ibrutinib, Treprostinil, Ambrisentan, and Macitentan) are predominantly indicated for hematologic malignancies, heart failure, or pulmonary hypertension. These underlying conditions or their disease management *per se* may independently increase the risk of hyperuricemia and gout attacks. For instance, patients with hematologic malignancies often exhibit high cell turnover or develop tumor lysis syndrome, leading to a sharp increase in uric acid production. In patients with heart failure or pulmonary hypertension, strict fluid management, diuretic use, or alterations in effective circulating volume may influence uric acid excretion or precipitate gout. Consequently, the observed associations between these drugs and gout may be confounded by indication—the underlying disease, rather than the drug (or the drug in synergy with the disease), could be the true driver of gout events. Although we applied LASSO regression to control for multiple covariates and conducted time-to-onset analyses suggesting that most events occurred within 0.6 years of drug initiation, the inherent limitations of the FAERS database (e.g., lack of detailed data on disease severity, concomitant medications such as diuretics or allopurinol/febuxostat use, and tumor lysis syndrome prophylaxis) preclude complete adjustment for these confounders. Therefore, the signals identified for these drugs should be considered hypothesis-generating rather than evidence of causal relationships. Future prospective cohort studies using large-scale real-world data (e.g., electronic health records) with rigorous matching or instrumental variable approaches are warranted to disentangle drug-specific effects from the intrinsic gout risk conferred by the underlying disease indications.

This pharmacovigilance analysis offers valuable evidence regarding the possible association between medication exposure and the onset as well as progression of gout; however, several limitations should be carefully considered. First, reliance on the FAERS spontaneous reporting system introduces inherent reporting biases, such as underreporting, duplicate entries, and variability in data completeness and quality, which may compromise the robustness of signal detection. Moreover, FAERS predominantly receives reports from the United States and a limited number of other countries, introducing potential regional bias; therefore, the findings may not be directly generalizable to populations with different genetic backgrounds, dietary patterns, urate-lowering treatment practices, or healthcare systems. Second, individuals with drug-associated gout frequently have coexisting comorbid conditions, and the residual confounding effects of these factors cannot be entirely controlled within the present analytical approach. Third, reports derived from passive surveillance systems are generally not subject to systematic validation; consequently, the observed associations should be interpreted as tentative pharmacovigilance signals rather than definitive evidence of causality. Accordingly, these findings primarily underscore signals that merit further exploration, and well-designed prospective investigations are required to substantiate the relationships observed.

## 5 Conclusion

In this investigation, a real-world pharmacovigilance framework was utilized to systematically examine potential pharmacological risk factors implicated in drug-related gout. Utilizing a comprehensive disproportionality analysis, 35 pharmaceutical agents were identified as potentially linked to the initiation and progression of gout. These results provide significant contributions to the early identification and risk stratification of medication-associated gout, offering evidence that may guide and refine clinical decision-making for at-risk patient populations. To confirm these associations and clarify the underlying pathophysiological mechanisms, further mechanistic research and well-designed prospective clinical trials are necessary.

## Data Availability

Publicly available datasets were analyzed in this study. This data can be found here: https://open.fda.gov/data/faers/.
